# Determinants of Hypovitaminosis D in Pregnant Women and Their Newborns in a Sunny Region

**DOI:** 10.1155/2013/460970

**Published:** 2013-07-01

**Authors:** Roya Kelishadi, Faezeh Sharifi-Ghazvini, Parinaz Poursafa, Ferdous Mehrabian, Sanam Farajian, Hosseinali Yousefy, Mahsa Movahedian, Sanaz Sharifi-Ghazvini

**Affiliations:** ^1^Pediatrics Department, Faculty of Medicine and Child Growth and Development Research Center, Isfahan University of Medical Sciences, Isfahan 81676-36954, Iran; ^2^Environmental Protection Department, Environment Research Center, Isfahan University of Medical Sciences, Isfahan 81676-36954, Iran; ^3^Obstetrics and Gynecology Department, Faculty of Medicine, Isfahan University of Medical Sciences, Isfahan 81676-36954, Iran; ^4^Department of Clinical Nutrition, School of Nutrition and Food Science, Isfahan University of Medical Sciences, Isfahan 81676-36954, Iran; ^5^Department of English Linguistics, The University of Isfahan, Isfahan 81676-36954, Iran; ^6^Faculty of Engineering, Ferdowsi University of Mashhad, Mashhad 9177948974, Iran

## Abstract

*Introduction.* This study aims to assess the factors associated with 25-hydroxy vitamin D (25(OH)D) levels in pregnant women and their newborns in a sunny region. *Materials and Methods.* This cross-sectional study was conducted in 2012 in Isfahan, Iran. It comprised 100 nulliparous singleton pregnant women, selected by random cluster sampling. Laboratory tests were assessed before delivery in mothers and after delivery in their infants' umbilical cord blood. The *P* for trend of variables was assessed across the air quality index (AQI) quartiles. The associations of AQI and 25(OH)D were assessed by multiple linear regression after adjustment for age, body mass index, and dietary intake. *Results.* Sera of 98 mothers and an equal number of newborns were analyzed. The median (interquartile range, IQR) of serum 25(OH)D of mothers and neonates was 15.1(12.6, 18.2) ng/mL in mothers and 15.7(12.0, 18.1) ng/mL in neonates, respectively. AQI had an inverse association with serum 25(OH)D (Beta = −0.58, *P* = 0.04). The corresponding figure was also inverse and significant for newborns (Beta (SE)= −0.51(0.04), *P* = 0.01). *Conclusion.* The independent inverse association of 25(OH)D with air quality can explain the high prevalence of hypovitaminosis D in pregnant women living in this sunny region.

## 1. Introduction

Vitamin D is one of the essential ingredients in terms of metabolic and physiological processes in the human body. The main source of vitamin D is its synthesis in dermis and epidermis, which is affected by ultraviolet B (UVB) rays, including approximately 90 percent of the entire body need. However, sources of this vitamin exist in foods such as egg yolk, fatty fish, fish oil, fortified foods and vitamin supplements [1], but diet provides only part of body needs. Thus, inadequate radiation or lack of UVB and in turn reduced dermal synthesis is considered as one of the main determinants of vitamin D deficiency [2]. Skin synthesis of vitamin D depends mainly on factors like: age, degree of skin pigmentation, and the extent of body attainable UVB, which are dependent on the amount of UVB radiation reaching the Earth's surface [3–6], which is in turn affected by geographic location, season, time, and level of atmospheric pollution [2, 3, 7–9]. 25-Hydroxyvitamin D[25(OH)D] is the most sensitive index of vitamin D status and reflective of its dietary intake and skin production. There are reports of varying degrees of vitamin D deficiency in women of countries with different geographical locations and various sociodemographic situations [10–18]. On the other hand, vitamin D has various health impacts, and its importance from early life is well documented [19, 20]. In a recent study in Isfahan, where the current study was conducted, low level of 25(OH)D was reported in 70.4% of the adult population; that is, 50.8% had vitamin D deficiency, and 19.6% had vitamin D insufficiency [21]. Different assumptions have been studied so far around the causes of vitamin D deficiency. Although the main source of vitamin D is its skin synthesis, most previous studies have focused on dietary intake of vitamin D, and limited experience exists about the role of environmental factors in the high prevalence of hypovitaminosis D. A study conducted in India found that the average level of serum 25(OH)D, in individuals living in a highly air-polluted area was 54% lower than those living in an area with low level of pollution [22]. Considering the importance of vitamin D in human body, especially in pregnant women and in turn in their infants, the present study was conducted in Isfahan as the second (and some days, the first) air-polluted city of Iran, to examine the association of air pollution with serum 25(OH)D in pregnant women and in the umbilical cord of their newborns.

The study comprised 100 pregnant women; demographic factors, dietary habits, and environmental factors as the ratio of ultraviolet (UV) and data of air quality index (AQI) were registered. Levels of 25(OH)D, parathyroid hormone (PTH), calcium (Ca), and albumin were measured before delivery in mothers and after delivery in their infants' umbilical cord blood. The *P* for trend of variables was assessed across the AQI quartiles. The associations of AQI and laboratory tests were assessed by multiple linear regression after adjustment for age, body mass index, and dietary intake.

## 2. Materials and Methods

This cross-sectional study was conducted in 2012 in Isfahan, Iran. It was approved by the Research Council and Ethics Committee of the School of Medicine, Isfahan University of Medical Sciences, Isfahan, Iran. It was conducted after obtaining written informed consent from participants. 

### 2.1. Study Area

 Isfahan is the second largest city in Iran located in the center of Iranian plateau, with a latitude of 32°37′N, longitude of 051°40′E, and an average altitude of 1500 m from the sea level bounded by NW-SE mountain range of 3000 m. It has a moderate and dry climate on the whole with sunny weather in most days throughout the year. It is an industrial city with a population of near two million; it is the second air-polluted city of the country. The air of this city is predominantly affected by industrial emissions and motor traffic [23].

### 2.2. Participants

 This study comprised 100 pregnant women and their newborns. Pregnant women were selected by random cluster sampling from among women referred to obstetrics clinics for the last visit before delivery. Those women who were eligible for the studies were healthy looking, nulliparous, and with singleton pregnancies and were living for at least one year in areas of the city with different levels of air pollution that had air pollution monitoring station. The neonatal umbilical cord blood of their healthy full-term newborns were studied as well. The demographic data such as mother's age, residence area, medications received during pregnancy, time of exposure to sunlight were recorded. Weight and height were measured with calibrated instruments (Seca, Japan) by using standard protocol. Mothers' body mass index (BMI) was computed as weight (Kg) divided by height squared (m^2^). Physical examination and measurement of weight, height, and head circumference of newborns were conducted by expert pediatricians under standard protocol and by using calibrated instruments.

### 2.3. Dietary Intake

 Nutrient intakes were estimated using a 3-day dietary record (two week days and one weekend day). Mothers were asked to complete the questionnaires, to write down the type and amount of food eaten, using scales or household measures to gauge portion sizes. Three-day averages of energy and macronutrient intakes were analyzed by the Nutritionist-4 software (First Databank Inc., Hearst Corp., San Bruno, CA). Data entry was performed by a trained dietitian. If a participant ate a food not included in the database, a food with very similar nutrient composition was selected. Nutrient information was also obtained through food labels or recipes from participants. Dietary intake was compared with the recommended dietary allowance (RDA) level during pregnancy, which is considered as 15 *μ*g [24].

### 2.4. Environmental Factors

Data from air pollution measurement stations in Isfahan city were recorded daily for the 7 days prior to blood sampling from participants. The air quality index (AQI) was registered. The mean values of seven 24-hour means of AQI were considered for statistical analysis. AQI provides a uniform system of measuring pollution levels for the major air pollutants. It is based on a scale devised by the United States Environmental Protection Agency (USEPA) to provide a way for broadcasting air quality on a daily basis. AQI of 0–50 is considered as good, 51–100 as moderate, 101–150 as unhealthy for sensitive groups, 151–200 as unhealthy, 201–300 as very unhealthy,and 301–500 as hazardous.

 We measured the ground levels of UVB by a Haze meter instrument, type UVB EC1 HANGER (Germany), between 9:00 and 12:00 am, in 8 areas in Isfahan. The unit of UVB measurements was watt per square meter (W/m^2^).

### 2.5. Laboratory Tests

After necessary accommodation, when pregnant mothers were referred to the laboratory for their routine pregnancy tests, the same blood sample was also used to determine 25(OH)D, parathyroid hormone (PTH), alkaline phosphatase (ALP), albumin, and calcium (Ca) levels. After delivery, a blood sample of newborn's umbilical cord was taken to determine the previously mentioned tests. All samples were centrifuged after 15 min incubation at room temperature. Sera level of 25(OH)D was measured using the chemiluminescent immunoassay (CLIA) method (25-OH D CLIA kit, DiaSorin, Stillwater, MN,USA); the kit expected range 4–150 ng/mL; the lowest reportable value was 4.0 ng/mL which is based on an interassay precision that approximates 20% CV (functional sensitivity). According to this kit, 25(OH)D levels less than 10 ng/mL and 10–30 ng/mL were considered as vitamin D deficiency and insufficiency, respectively.

Sera PTH level was determined using an chemiluminescent immunnoassay (CLIA) (CLIA kit, DiaSorin, USA). Ca, P, and total ALP levels were measured using photometric methods (Calcium CPC, Phosphate UV, and Alkaline Phosphatase DGKC Kit, Pars Azmoon Co., Tehran, Iran). The sensitivity of assays mentioned was 0.2 mg/dL, 0.7 mg/dL and 3 U/L, respectively. Intra- and interassay CV% for calcium, and total ALP of the samples were 1.4, 2.7, 1.9, 3.1, and 1.1, 1.8, respectively. 

### 2.6. Statistical Analysis

Data analyses were performed using SPSS (version 16 : 0, SPSS Inc., Chicago, IL) software. The normality of the distribution of variables was confirmed by Kolmogorov-Smirnov test. Continuous variables are reported as the mean ± SD. The AQI levels were categorized to quartiles, and the upper quartile was considered as elevated level. The *P* for trend was assessed for different variables across the AQI quartiles. The associations between AQI and laboratory tests were assessed by multiple linear regression after adjustment for age, BMI, and dietary intake. *P* value of <0.05 was considered as statistically significant.

## 3. Results

Sera of 98 mothers and an equal number of their neonates were included in the statistical analysis.  Table 1 presents the median (IQR) of variables studied among mothers and newborns. The median of 25(OH)D of both mothers (15.1 ng/mL) and newborns (15.7 ng/mL) was low. The vitamin D intake of mothers was 11.9 *μ*g, which corresponded to 79% of RDA. The median AQI was 144.1, that is, unhealthy for sensitive groups, and its range varied to unhealthy levels for general population.

As presented in Table 2, the median (IQR) of ultraviolet B radiation and serum 25(OH)D of mothers and neonates in areas with the highest pollution (AQI Quartile4) was significantly lower than in those living in areas with the lowest pollution level (AQI Quartile1). Both in mothers and neonates, the median (IQR) of PTH was higher in area with the highest air pollution than in area with the lowest air pollution level.

Multiple regression analysis showed that AQI had an inverse association (Beta = −0.58, *P* = 0.04) with serum 25(OH)D, which remained significant after adjustment for age, BMI, and dietary intake. The corresponding figure was also inverse and significant for newborns after adjustment for their gender and anthropometric measurements (Beta (SE) = −0.51(0.04), *P* = 0.01).

## 4. Discussion

This study revealed low levels of 25(OH)D in mothers and neonates living in a sunny region with moderate dietary intake of vitamin D by pregnant mothers. The air quality had an inverse and independent association with 25(OH)D levels of mothers and their neonates. Hypovitaminosis D of pregnant women is considered as a global health problem. A recent population-based study on pregnant women of multiethnic groups confirmed the high prevalence of vitamin D deficiency and insufficiency year-round among pregnant women in North West London, especially among those who had darker skin [25]. It is well documented that newborns receive their vitamin D completely from the vitamin D stores of their mothers, and after birth their vitamin D stores are about two-thirds of maternal levels [26]. Vitamin D deficiency of pregnant women has several adverse health effects, not only for themselves [27, 28], but also for their children [29, 30]. Recent studies in Turkey, our neighbor country with climate, culture, and dietary habits very similar to Iran, showed very low levels of 25(OH)D in pregnant women and their newborns [31, 32]. 

Although these studies suggested the life style and nutritional status of mothers as the etiology of hypovitaminosis D, we suggest that the role of environmental factors, notably air pollution, should also be considered in this regard.

 Limited numbers of studies have been done on environmental factors affecting hypovitaminosis D. Air pollution is one of the main causes of Earth's surface UVB determination. A study conducted in India showed that the atmospheric pollution causes a reduction in the percentage of Earth's surface UVB and children in high polluted areas are at higher risk for vitamin D deficiency [22]. In another study done in Belgium on menopausal women, a direct relationship was found between air pollution and hypovitaminosis D [23]. A study in two cities of Iran, showed lower prevalence of hypovitaminosis D in women living in a small city than in those living in metropolitan Tehran. It suggested the independent role of air pollution in vitamin D deficiency among women [33]. Factors that affect cutaneous synthesis of vitamin D include use of sunblock, levels of sunlight exposure (such as season, latitude, and time of day), and also skin pigmentation [34]. A newborn's 25(OH)D concentration is approximately one-half that of his/her mother [35]. A risk factor for low 25(OH)D level in early infancy is maternal vitamin D deficiency during pregnancy, resulting in inadequate maternal transfer of vitamin D to the fetus and low infant stores [36–38]. In addition, skin synthesis of vitamin D, mediated by UVB, provides 90% of the body's need, and severe vitamin D deficiency causes rickets, which leads to permanent deformity of bones in children and osteomalacia and muscle weakness in adults. Transplacental passage of maternal 25(OH)D is the only source of vitamin D. Accordingly, pregnant women need to be vitamin D replete at the time of giving birth to ensure sufficient levels of this vitamin in their baby to last the first 4–6 months of life. Hypovitaminosis D during pregnancy and in turn neonatal period and infancy is of special concern [39–43]. Serum 25(OH)D levels and air quality may affect fetal growth [44, 45]. However, the present study showed no significant difference in variables of weight and height in newborns among quarters of air quality. This could be due to the existence of air pollution in all areas studied. In fact, there was no possibility to compare polluted and clean air. A study among Chinese population demonstrated that newborns of mothers with severe vitamin D deficiency had lower birth length and weight, with lower head circumference and birth weight in vitamin D-deficient newborns than in other neonates [46]. We did not document such difference, and this might be because 25(OH)D was low among most mothers and neonates, and it was not possible to compare newborns with low and adequate 25(OH)D levels. A recent study revealed that obese women transfer less 25(OH)D to their offspring than normal-weight women. Cord blood 25(OH)D levels have a direct correlation with the neonatal percentage of body fat [47]. In our study, we adjusted the statistical analysis for anthropometric measures of mothers and neonates, and we documented the independent inverse association of air quality with 25(OH)D levels in pregnant mothers and their neonates. Given the importance of vitamin D and the high prevalence of hypovitaminosis D, many experts recommend routine supplementation of vitamin D from early life [48]. A study in 2012 reviewed all clinical studies published in the previous 15 years regarding vitamin D deficiency in pregnancy and its effects on their offspring and the vitamin D supplementation during pregnancy. It revealed that several studies have demonstrated the association of 25(OH)D deficiency with important health outcomes in the preconception period, during pregnancy, in perinatal period and in childhood. It suggested that 25(OH)D concentration of >32 and <50–60 ng/mL may be associated with the lowest risk of disease. Some studies had proposed to administer 2,000 IU/day of vitamin supplement to pregnant and breastfeeding women; however, some others suggested that 400–600 IU/day is enough [49]. However, as another systematic review and meta-analysis suggested U-shaped associations of vitamin D intake in pregnancy with extraskeletal health in children [50], supplementation of vitamin D and its doses warrants caution. Our findings may serve as confirmatory evidence of the necessity of high dose vitamin D intake of pregnant women living in industrialized and air-polluted areas as well as the importance of vitamin D supplementation of infants even in sunny regions. Considering industrial and developing conditions of the country, our findings suggest that one of the priorities of the health system is a special attention to the problem of air pollution, which needs action-oriented strategies. Moreover, it is suggested to increase the awareness of people, especially women, about the factors affecting vitamin D status. These factors include consuming rich sources of vitamin D, exposure to sunlight, and also emphasizing the prescription of vitamin D supplements to infants, even in sunny areas.

## Figures and Tables

**Table 1:** General characteristics of variables studied.

	Median	Interquartile range
Newborns		
Weight (gr)	3105.0	2975.0–3780.0
Length (cm)	49.1	48.5–51.5
Head circumference (cm)	34.5	33.8–35.1
25(OH)D (ng/mL)	15.7	12.0–18.1
Calcium (mg/dL)	9.6	8.8–10.7
Alkaline phosphatase (U/L)	361.0	350.5–392.0
Parathormone (Pg/mL)	24.0	21.5–27.0
Albumin (g/dL)	3.8	3.5–4.6
Mothers		
25(OH)D (ng/mL)	15.1	12.6–18.2
Calcium (mg/dL)	9.8	8.4–10.5
Alkaline phosphatase (U/L)	364.5	351.0–386.5
Parathormone (Pg/mL)	54.0	46.5–59.0
Albumin (g/dL)	3.9	3.6–4.5
Mothers' dietary intake		
Calcium intake (mg/day)	1014.71	875.0–2075.11
Daily dietary vitamin D intake (*μ*g)^a^	11.9	10.1–14.2
Environmental factors of mothers' living area		
Air quality index^b^	144.1	135.0–177.0
Ultraviolet B (W/m^2^)	0.41	0.35–0.44

^a^0.025 *μ*g = IU; ^b^air quality index (AQI): 0–50: good, 51–100: moderate, 101–150: unhealthy for sensitive groups, 151–200: unhealthy, 201–300: very unhealthy, and 301–500: hazardous.

**Table 2:** Variables studied according to the quartiles of the air quality index.

	Quartile 1	Quartile 2	Quartile 3	Quartile 4	*P* for trend
Air quality index	137.0 (135.0, 139.0)^a,b^	140.5 (138.7, 150.0)	145.0 (139.0, 166.2)^b^	149.5 (145.8, 177.0)^a^	0.02
Ultraviolet B (W/m^2^)	0.41 (0.39, 044)^a,b^	0.39 (0.37, 0.40)	0.37 (0.35, 0.39)^b^	0.36 (0.35, 0.38)^a^	0.04
Newborn weight (gr)	2980.0 (2975, 3100)	3000 (2970, 3250)	2990 (2970, 3365.0)	2950 (2980, 3350.0)	0.6
Newborn length (cm)	49.4 (48.8, 51.1)	49.7 (48.9, 50.4)	49.3 (48.8, 51.4)	49.7 (48.7, 51.1)	0.8
Newborn 25(OH)D (ng/mL)	16.9 (14.0, 21.1)^a,b^	16.1 (14.2, 21.4)	15.7 (14.8, 17.1)^b^	15.1 (14.0, 17.7)^a^	0.03
Newborn parathormone (Pg/mL)	22.1 (21.5, 23.4)^a^	22.7 (21.9, 24.1)	23.8 (22.7, 24.1)	24.2 (22.4, 27.0)^a^	0.04
Mothers' 25(OH)D (ng/mL)	16.5 (15.1, 18.2)^a,b^	16.0 (14.8, 19.1)^c^	14.5 (13.1, 17.9)^b^	13.8 (13.7, 15.1)^a,c^	0.04
Mothers' parathormone (Pg/mL)	48.1 (46.5, 50.7)^a^	51.8 (48.7, 53.2)^c^	53.2 (51.1, 55.7)	54.0 (52.8, 59.0)^a,c^	0.04

Data presented as median (interquartile range); the dietary intake of calcium and vitamin D was compared in various AQI quartiles and had no significant difference.

^a^ *P* < 0.05 between the first and fourth quartiles; ^b^ *P* < 0.05 between the first and third quartiles; ^c^ *P* < 0.05 between the second and fourth quartiles; *P* < 0.05 between the second and third quartiles.

Air quality index (AQI): 0–50: good, 51–100: moderate, 101–150: unhealthy for sensitive groups, 151–200: unhealthy, 201–300: very unhealthy, and 301–500: hazardous.
